# Metabolic Reprogramming and Immune Evasion in Nasopharyngeal Carcinoma

**DOI:** 10.3389/fimmu.2021.680955

**Published:** 2021-09-09

**Authors:** Huimei Huang, Shisheng Li, Qinglai Tang, Gangcai Zhu

**Affiliations:** Department of Otolaryngology-Head and Neck Surgery, The Second Xiangya Hospital, Central South University, Changsha, China

**Keywords:** nasopharyngeal carcinoma, metabolism, immune cell, pH, immunotherapy

## Abstract

Nasopharyngeal carcinoma (NPC) is a malignant tumor of the nasopharynx mainly characterized by geographic distribution and EBV infection. Metabolic reprogramming, one of the cancer hallmarks, has been frequently reported in NPCs to adapt to internal energy demands and external environmental pressures. Inevitably, the metabolic reprogramming within the tumor cell will lead to a decreased pH value and diverse nutritional supplements in the tumor-infiltrating micro-environment incorporating immune cells, fibroblasts, and endothelial cells. Accumulated evidence indicates that metabolic reprogramming derived from NPC cells may facilitate cancer progression and immunosuppression by cell-cell communications with their surrounding immune cells. This review presents the dysregulated metabolism processes, including glucose, fatty acid, amino acid, nucleotide metabolism, and their mutual interactions in NPC. Moreover, the potential connections between reprogrammed metabolism, tumor immunity, and associated therapy would be discussed in this review. Accordingly, the development of targets on the interactions between metabolic reprogramming and immune cells may provide assistances to overcome the current treatment resistance in NPC patients.

## Introduction

Nasopharyngeal carcinoma (NPC) is a unique epithelial malignancy with the highest incidence in distinctive ethnic and geographic populations in Southeast Asia, and it is characterized by lymphoepithelial-like histological features and Epstein-Barr virus (EBV) infection (1). More than 70% of the 130,000 new global cases reported each year are diagnosed in East and Southeast Asia, especially southern China (2). GBD Diseases and Injuries Collaborators reported that, by 2019, the fatality rate and disability-adjusted life years of patients with NPC had increased annually (3). Although the 5-year overall survival rate in patients with early-stage NPC is approximately 95%, it is only 54.2% in early-staged NPC patients (4). Notably, the nasopharynx, enriched with lymphoid tissues, is a unique inductive site for B cell responses, plasma cell generation, and naïve T cell maturation and differentiation into the effector and memory T cells that eliminate inciting antigen cells, mounting a defense against NPC initiation and development (5). The mechanism by which NPC cells avoid immune surveillance remains unclear. Somatic alternations in chromatin modification and metabolism-associated genes, identified by two whole-exome sequencing studies (6, 7), imply the importance of reprogramming energy metabolism in NPCs. Reprogramming energy metabolism and evading immune destruction have been extensively investigated in NPC (8). Recently, emerging strategies have been avidly explored to target metabolic pathways that enhance anti-tumor immunity. However, no review has focused on these two features or their mutual connections in NPC; here, we review the dysregulated metabolic processes, including glucose, fatty acid, amino acid, and nucleotide metabolism, and the mutual interplay of these processes in NPC. Moreover, the potential connections between reprogrammed metabolism, tumor immunity, and the associated therapies are discussed in this review.

## Aerobic Glycolysis

**Figure 1:**
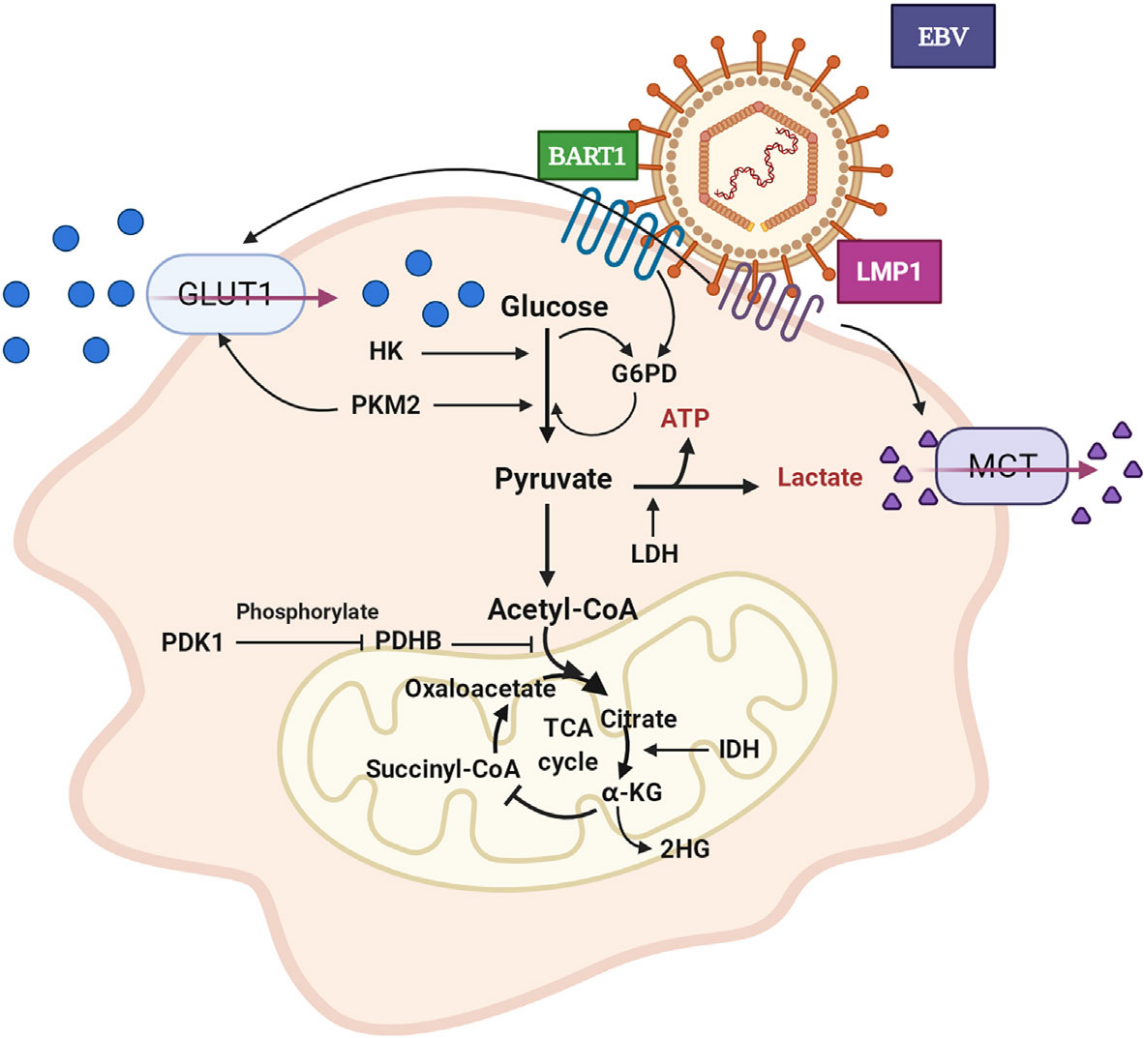
Aerobic glycolysis of NPC cell. GLUT1 expression is up-regulated and glucose uptake is increased. The expression level of HK is increased and promotes glycolysis. PKM2 over-expression promotes GLUT1 expression and glucose absorption. The expression of PDHB is down-regulated, which inhibits the conversion of pyruvate to acetyl CoA. PDK1 is highly expressed, which leads to the inactivation of PDHB. The over-expression of IDH2 promoted the transformation of α - KG to 2-HG. LDH expression and lactate production increased. EBV secreted-protein LMP1 promotes glucose uptake and lactate production. In pentose phosphate pathway, EBV-miR-BART1 promotes G6PD over-expression. GLUT1: glucose transporter 1, HK: hexokinase, PKM2: pyruvate kinase M2, PDHB: pyruvate dehydrogenase B, PDK1: pyruvate dehydrogenase kinase 1, IDH2: isocitrate dehydrogenase, α-KG: α–ketoglutarate, 2-HG: 2-hydroxyglutaric acid, LDH: lactate dehydrogenase, LMP1: EBV latent membrane protein 1, BART1: EBV-encoded microRNA BART1, G6PD: glucose-6-phosphate dehydrogenase.

In correlative studies of tumor metabolism, the earliest and most focused point is the Warburg effect, through which cancer cells consume glucose to produce lactate even when the oxygen level is sufficient and do not enter the TCA cycle (9). The glucose metabolism of NPC (as shown in **Figure 1**) mostly depends on aerobic glycolysis, and it is frequently accompanied by the abnormal expression of glucose transport and metabolic enzymes (hexokinase (HK), phosphofructokinase-1, lactate dehydrogenase (LDH), etc.) (10).

### GLUT1 for Glucose Transport

The application of [18F]-fluorodeoxyglucose positron emission tomography (PET-CT) in NPC patient imaging in clinical practice has provided the best evidence to support the increased uptake of glucose in NPC (11). Several investigations have shown that total lesion glycolysis (TLG), a prognostic indicator, is in consistent with metabolic tumor volume (MTV) NPC patients, as shown by [F18]-PET-CT (11–13). GLUT1 has a high affinity for glucose, facilitating glucose uptake from outside cancer cells (14). GLUT-1 expression is relevant to clinical stage and lymph node metastasis in NPC (15). EBV exists in almost all NPCs. LMP1, an EBV-encoded specific protein, promotes GLUT-1 transcription by activating mTORC1/NF-κB signalling, resulting in the regulation of aerobic glycolysis and the growth of NPC cells (16). Effective inhibitors of the GLUT family can inhibit glycolysis flux and tumor growth (17). Regulatory enzymes of glucose metabolism such as pyruvate kinase affect the process of GLUT1-induced glucose transportation. Aberration of M2 pyruvate kinase (PKM2) is notable in NPC cells. Inhibition of PKM2 decreases GLUT1 activity and the Warburg effect in NPC cells (18). As summarized in **Table 1**, GLUT1 and PKM2 show hypomethylation in their promoter regions in NPC.

**Table 1:** The Aberrant Methylation of the Key Metabolic Genes in NPC Microarrays.

Genes	Differential probes	Region	Consequence
GLUT1	Cg03106288	1^st^ exon; 5’UTR	Hypomethylation
PKM2	Cg16940801	1^st^ exon; 5’UTR	Hypomethylation
IDH1	Cg10356455	TSS200	Hypomethylation
FASN	Cg14521508	TSS200	Hypomethylation

*Differential probes were the overlap of E-GEOD-62336 and E-GEOD-52068.

### From Glucose to Pyruvate

Aerobic and anaerobic oxidation of glucose proceed through three and two stages, respectively. These processes share the same glycolysis stage in which glucose is decomposed into two molecules of pyruvate. Three vital enzymes, hexokinase (HK) (also known as glucokinase), phosphofructokinase 1 (PFK1), and pyruvate kinase (PK), determine glycolytic flow speed and direction (19). HK is associated with NPC patient prognosis and outcomes, and its expression has been shown to be promoted by FOXC2-YAP signalling in NPC cells (20, 21). HK inhibitors can limit tumor glycolysis and increase free radicals that result in cell apoptosis (22, 23). The abnormal expression of PK enhances glucose uptake and increases the rate of phosphoenolpyruvate to pyruvate (18). Pyruvate kinase M2 (PKM2), a subtype of PK, is overexpressed in NPC cells and upregulated significantly by activation of the phosphatidylinositol 3 kinase (PIK3)/AKT signalling pathway, leading to increased glucose uptake, lactate, and ATP levels (24). In addition, PKM2 promotes cancer metastasis by activating EGFR-stimulated nuclear translocation of PKM2, and PKM2 regulates the transcription of FOSL1 and ANTXR2 (25). Ten-eleven translocation protein 2 (TET2) can block the process of PKM nuclear translocation and thus inhibit the glycolysis, proliferation, and invasion of NPC cells (26). To date, PFK1 has not been reported in NPC. However, early studies revealed that upregulation of hypoxia-inducible factor 1α (HIF-1α) in carcinoma can increase PFK1 protein expression and promote aerobic glycolysis, providing some clues for future NPC research (27, 28). Taken together, the regulation of these key enzymes in NPC cells may control aerobic glycolysis and lead to NPC development.

### From Pyruvate to ATP and Carbon Dioxide in Mitochondria

In the second stage of aerobic oxidation, pyruvate is transported to mitochondria where it is converted to acetyl-CoA. The reaction that converts pyruvate into acetyl-CoA is catalysed by the pyruvate dehydrogenase complex (PDHC), which includes pyruvate dehydrogenase (E1), dihydrolipoamide transacetylase (E2), and dihydrolipoamide transdehydrogenase (E3) (19). Previous studies have found that the expression of pyruvate dehydrogenase B (PDHB, a member of PDHC-E1) was downregulated in the context of NPC, which inhibited the conversion of pyruvate to acetyl-CoA (29). In addition, genes related to PDHB are abnormally expressed in cancer stem cell-like cells (30). Moreover, PDH action is regulated by PDK. PDK1 is a pyruvate dehydrogenase kinase isoenzyme located in the mitochondria and overexpressed in NPC cells, and it mediates the conversion of pyruvate to acetyl-CoA, inactivates PDH, playing an important role in the regulation of glucose and fatty acid metabolism. The frequent overexpression of PDK1 in primary NPC is associated with poor patient prognosis, clinical stage, and metastasis (31, 32). Chibby, a β-Catenin-related antagonist, has been shown to arrest aerobic glycolysis in human NPC *via* inhibition of PDK1 (33). In addition, in head and neck squamous cell carcinoma, mitochondrial gene mutations promote the accumulation of HIF-1α because PDH is downregulated by PDK2 (34). In general, PDK exerts a crucial influence on the metabolic regulation of NPC.

In the third stage of aerobic oxidation, acetyl-CoA enters the TCA cycle and is coupled with oxidative phosphorylation to form ATP. This process requires catalysis of at least seven metabolic enzymes, of which the three most important rate-limiting enzymes are citrate synthase (CS), isocitrate dehydrogenase (IDH), and the α-ketoglutarate dehydrogenase complex (α-KGDH) (19). Isocitrate dehydrogenase 1 (IDH1) exhibits hypomethylation in NPC (**Table 1**). Isocitrate dehydrogenase 2 (IDH2) is involved in EBV-dependent metabolic reprogramming and tumorigenesis. The expression of IDH2 is upregulated in NPC, leading to increased intracellular α-ketoglutaric acid (α-KG)-catalysed 2-hydroxyglutaric acid (2-HG). The level of 2-HG is positively correlated with local lymph node metastasis in NPC (35). However, recent studies have found that IDH2 is differentially mutated in different NPC subtypes (36, 37). IDH mutation endows the enzyme product with the novel ability to catalyse α-ketoglutaric acid (α-KG) to 2-hydroxyglutaric acid (2HG). Ultimately, it can be inferred that IDH blocks the TCA cycle by catalysing the conversion of α-KG to 2HG. Unfortunately, the roles of the other two rate-limiting enzymes, α-KGDH and CS, have not been reported in NPC. However, mounting evidence suggests that high expression of succinate dehydrogenase B (SDHB) in recurrent local NPC is beneficial for prolonging patient survival time, indirectly implying roles for α-KGDH and CS in NPC cells (38).

### From Pyruvate to Lactate and the Pentose Phosphate Pathway

Lactate is the largest glucose metabolite in carcinoma tissue. The reduction of pyruvate to lactate, which is crucial to the Warburg effect in cancer cells, is catalysed by lactate dehydrogenase (LDH). High serum LDH levels are associated with poor survival in NPC (39). Inhibition of LDH can induce G2/M cell cycle arrest by downregulating the CDK1/cyclinB1 pathway, consequently promoting cell apoptosis by increasing mitochondrial ROS production (40). The expression of lactate dehydrogenase 5 (LDH-5) is also increased in oral squamous cell carcinoma (OSCC) (41). Currently, LDH-5, a subtype of lactate dehydrogenase, is considered a promising anticancer target (42). Hence, efforts to reprogramme LDH have led to a significant change in the production of lactate.

*The pentose phosphate* pathway is another pathway of glucose metabolism that substantially contributes to biosynthesis and antioxidation. The most important catalytic enzyme in the *pentose phosphate* pathway is glucose-6-phosphate dehydrogenase (G6PD). G6PD participates in the metabolic pathway of pentose phosphate to produce NADPH and ribose phosphate (43). Through oxidation and group transfer, fructose-6-phosphate and glyceraldehyde 3-phosphate are formed and return to the glycolysis metabolic pathway. NADPH activity induced by G6PD can maintain reduced levels of glutathione, which has been found to be an important antioxidant *in vivo* (44). NPC patients with low serum levels of G6PD tend to relapse and have a poor prognosis. To promote tumor cell antioxidation and prevent oxidative damage, increased expression of G6PD may be attenuated by EBV-miR-BART1 signalling in NPC cells (45, 46).

### Monocarboxylic Acid Transporter

Lactate produced by cancer cells is conveyed to the extracellular environment through monocarboxylic acid transporters (MCTs), especially monocarboxylic acid transporter 4 (MCT4), thus forming an acidic tumor microenvironment (47). Moreover, cancer cells within the tumor cooperate to establish metabolic “symbiosis”. Cancer cells in hypoxic regions consume glucose and release lactate through anaerobic glycolysis, and similar to a lactate shuttle, this lactate is used as fuel for the TCA cycle by cells in adjacent oxygen-containing tumor areas (48). This movement of lactate shows interplay with monocarboxylic acid transporters (MCTs), which are differentially expressed: Anoxic cancer cells overexpress MCT4 as the main lactate export transporter, and oxygen-rich cancer cells overexpress MCT1 as a lactate input transporter (49). MCT1 is highly expressed in NPC and may promote cell invasion and migration through the PI3K/Akt signalling pathway (50).

### Effect of LMP1 on Aerobic Glycolysis

EBV latent membrane protein 1 (LMP1) can promote aerobic glycolysis in NPC cells. LMP1 significantly increases glucose consumption, lactate production, and LDH activity. The content of hypoxia-inducible factor 1 (HIF-1α) and that of its targets PDK1 and PKM2 also continuously increase (51). LMP1 activates a variety of cellular signalling pathways (52). Studies have suggested that LMP1 is associated with EBV-driven NPC pathogenesis by mediating the FGF2/FGFR1 signalling pathway. It increases glucose and glutamine uptake, LDHA activity, and lactate production by activating the FGFR1 signalling pathway and increases the phosphorylation of PKM2 and LDHA, as well as the expression of PDHK1, c-Myc, and HIF-1α (53). In summary, these findings suggest that LMP1 promotes aerobic glycolysis by regulating changes in metabolic enzymes and related genes such as c-Myc and HIF-1α.

## Fatty Acid Metabolism

### Lipid Droplet Transport

The high demand for nutrients in tumor cells increases the uptake and synthesis of fatty acids. In addition to the *de novo* synthesis of fatty acids, dietary fatty acids in the blood are decomposed into triglycerides by lipoprotein lipase (LPL) and transported into tumor cells by CD36 (a fatty acid transferase) (54, 55). Serum lipid levels constitute a risk factor for cancer metastasis. In contrast to the level in normal nasopharyngeal epithelium, the accumulation of lipid droplets in NPC increases greatly, which may be associated with downregulation of the lipolysis gene adipose triglyceride lipase (ATGL) *via* EBV-encoded LMP2A (56). Lipid-lowering drugs, such as lovastatin, can effectively inhibit nasopharyngeal oncogenesis (57, 58).

### Fatty Acid Synthesis

Under normal circumstances, the sources of fatty acids are mainly exogenous fatty acids digested and absorbed in the small intestine and then synthesized in the liver. However, malignant cells, including NPCs, can independently synthesize endogenous fatty acids due to the high expression of fatty acid synthase (FASN) (59, 60). As shown in **Table 1**, FASN is hypomethylated in the TSS200 region. Silencing or targeting FASN makes NPC cells more sensitive to radiation (61, 62). In addition, quercetin, a polyphenolic flavonoid interferes with the activity of FASN and reduces NPC cell proliferation (63). Consistently, the expression of fatty acid synthase-related protein (FADD) in nasopharyngeal cells has been found to be higher than that in noncancerous tissues (64). Accordingly, acetyl-CoA transported to the cytoplasm *via* the citric acid-pyruvate cycle allows NPC cells to synthesize fatty acids *de novo* and provides rich substrates for the β-oxidation of fatty acids. Malic enzyme 1 (ME1) overexpressed in NPC cells catalyses the malic acid to pyruvate conversion and then promotes NADPH production to satisfy the increasing demand for *de novo* fatty acid synthesis (65).

### Fatty Acid Oxidation

β-oxidation of fatty acids (FAO) produces a large amount of ATP to meet cellular needs. Fatty acids are oxidized into acyl CoA and then transported to mitochondria by carnitine acyltransferase 1 (CPT1). Acetyl-CoA, FADH2, and NADH are formed by stepwise processes involving dehydrogenation, hydration, dehydrogenation, and thiolysis in mitochondria (19). CPT1 is a rate-limiting enzyme that affects the role played by acyl-CoA in mitochondria. Studies suggested that FAO is highly activated due to the upregulated expression of CPT1A in radiation-tolerant NPC cells, which supplies energy for the proliferation and progression of tumor cells (66). PROX1 promotes β-oxidation of fatty acids by upregulating CPT1A expression, and acetyl-CoA cooperates with histone acetyltransferase p300 in the acetylation of histones on lymphangiogenic genes. Thus, the PROX1-p300 interaction facilitates preferential histone acetylation (67, 68). This indicates that CPT1A may mediate epigenetic changes in oncogenes by enhancing fatty acid availability for acetyl-CoA. Taken together, fatty acid β-oxidation can promote the proliferation and differentiation of lymphatic endothelial cells, repair pathologically damaged lymphatic vessels, and contribute to favourable conditions for lymphatic metastasis of malignant cells. Thus, blocking CPT1A activity inhibits lymphangiogenesis, implying a potential therapeutic role for targeting FAO in NPC lymphatic metastasis (67, 68). To prevent lipid droplet accumulation and leverage this reduced accumulation, SOD1, a regulator of antioxidants, is highly expressed in NPCs. Therefore, blocking the activity of SOD1 can cause lipid accumulation and probably inhibit NPC cell growth (69). When the energy demand of tumor cells increases, peroxisome proliferator-activated receptor coactivator 1 (PGC-1α) can promote FAO in NPC cells *via* the CEBPB/CPT1A signalling axis to protect mitochondria from toxic lipid overload and resist radiation (70). Monoacylglycerol lipase (MAGL) is a serine hydrolase that can hydrolyse monoacylglycerol esters into free fatty acids and glycerol (71). MAGL is highly expressed in NPC and may affect CPT1-mediated β-oxidation through the FFA pathway, promote the oxidative decomposition of fatty acids, and promote metastasis through the epithelial-mesenchymal transition (EMT) (71, 72). Moreover, the CPT1A expression level is significantly correlated with the prognosis of NPC patients.

The acetyl-CoA produced by fatty acid β-oxidation is partially converted into ketone bodies (73). Inhibition of ketone body metabolism has been found in NPC in recent years. HMG-CoA lyase (HMGCL), a vital enzyme in the metabolic pathway of ketone bodies, is inactivated in NPC cells, which boosts their proliferation and metastasis by reducing the production of β-hydroxybutyric acid (β-HB) and inhibiting reactive oxygen species (ROS) generation (74). Therefore, agents that target pathways that regulate the homeostasis of fatty acid metabolism in NPCs, such as Pueraria lobata extract, may be effective because they disrupt lipid metabolic reprogramming (75, 76).

**Figure 2:**
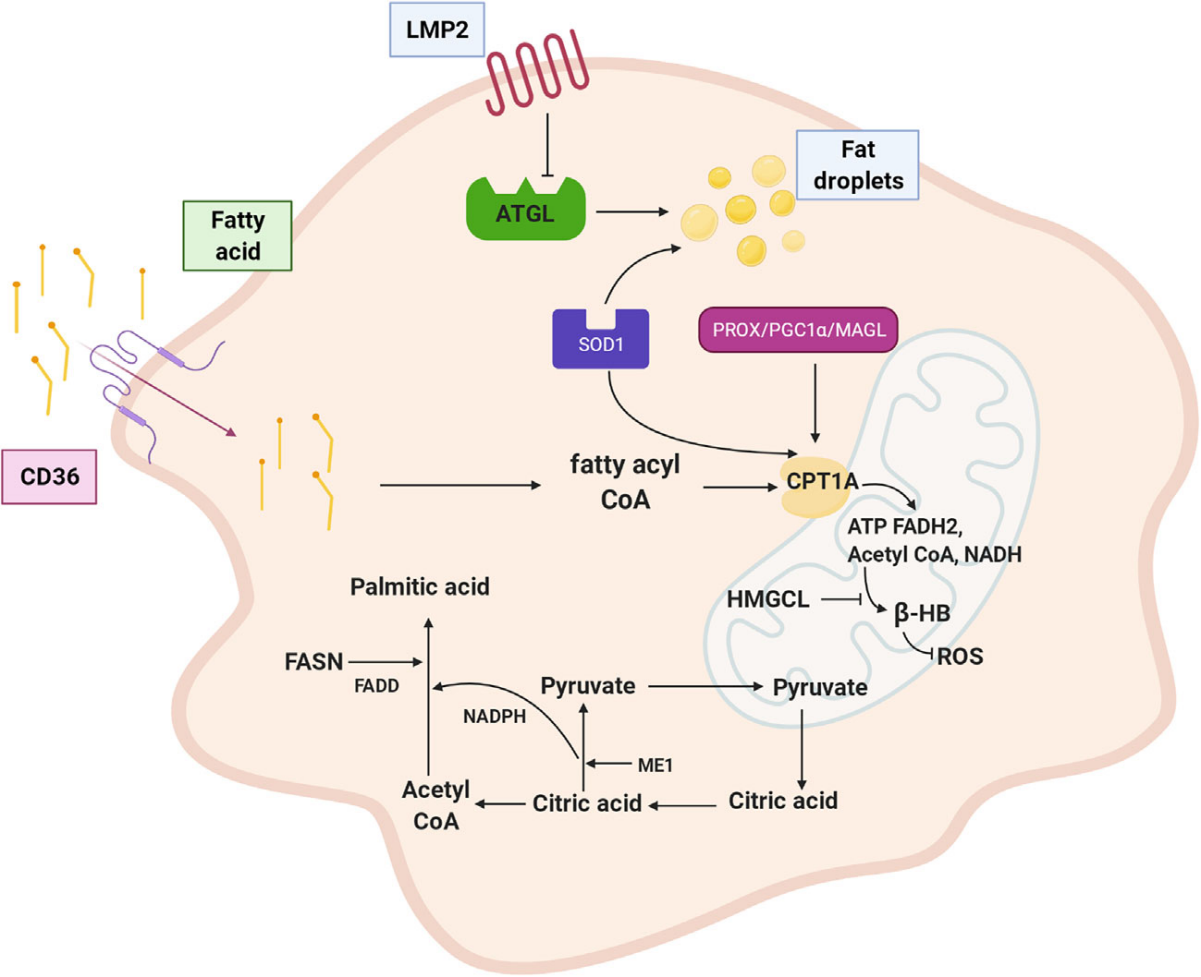
Fatty acid metabolism of NPC cell. LMP2A down-regulates lipolysis gene ATGL and promotes fat droplet accumulation; CD36 transports fatty acid to the cytoplasm. ME1 enhances malate pyruvate cycle, promotes transfer of acetyl CoA from mitochondria to cytoplasm, and participates in palmitic acid synthesis. FASN is up-regulated expression and palmitic acid synthesis is vigorous. CPT1A is positively regulated by PROX1、SOD1、PGC1α and MAGL. Inactivation of HMGCL inhibits ketone like β-HB metabolism of fatty acid and reduced production of ROS. LMP2A: EBV encoded latent membrane protein 2A, ATGL: adipotriglyceride lipase, CD36: fatty acid translocase, ME1: malic enzyme 1, FASN: fatty acid synthase, CPT1A: carnitine acyltransferase 1, PROX1: Prospero homeobox protein-1, SOD1: superoxide dismutase 1, PGC1α: peroxisome proliferator-activated receptor coactivator 1, MAGL: monoacylglycerol lipase, HMGCL: HMG-CoA lyase, β-HB: β-hydroxybutyric acid,ROS: reactive oxygen species.

In conclusion, lipid metabolism changes in NPC include alterations to lipid transport and absorption, fatty acid synthesis, and fatty acid oxidation. As shown in **Figure 2**, LMP2A encoded by EBV downregulates ATGL to accumulate lipid droplets; CD36 transports lipids to the cytoplasm. The malate-pyruvate cycle and transfer of acetyl-CoA from mitochondria to the cytoplasm for fatty acid synthesis are accelerated by ME1. FASN notably upregulates palmitic acid expression, catalysing palmitic acid biosynthesis to produce free palmitic acid. CPT1A is regulated by various genes (PROX1, SOD, PGC1α, and MAGL), leading to active fatty acid oxidation in mitochondria, which supplies abundant ATP and acetyl-CoA for the body and meets the material and energy needs of proliferating, growing, and metastasis of NPC cells. Furthermore, the metabolism of fatty acids into ketone bodies is inhibited by inactivated HMGCL and reduced ROS levels.

## Amino Acid Metabolism

Exogenous proteins are hydrolysed into amino acids and oligopeptides in the stomach and small intestine and then absorbed by different transporters or in the γ-glutamyl cycle. The activation of corresponding α-ketoacids, induced by glucose metabolism or amino acid catabolism, generates certain nonessential amino acids (alanine, aspartic acid, and glutamic acid). A deaminated amino acid can be converted to α-keto acid, while the coenzyme pyridoxal phosphate is converted to pyridoxamine phosphate and transfers amino groups to the corresponding residue. The excess ammonia produced during amino acid metabolism enters the urea cycle and excreted as urea out of the body (19). Arginine succinate synthase (ASS1) is a key enzyme of urea metabolism that inhibits the removal of ammonia in NPC (77).

Glutamine provides carbon and nitrogen for biosynthesis (78). Glutamine synthetase catalyses the conversion of glutamate with ammonia into glutamine (79). Glutamine synthetase genes are highly expressed in radiation-resistant cancer cells, promoting recovery of malignant cells from G2/M blockades (80). This may be the mechanism by which NPC cells exhibit resistance to radiotherapy. Moreover, the expression of GLS1-encoded glutaminase K (KGA) and glutaminase C (GAC) is increased in EBV-infected NPC cells. In addition, the expression of glutamate dehydrogenase 1 (GLUD1) and glutamate dehydrogenase 2 (GLUD2) is also upregulated in NPC cells, and they catalyse the conversion of glutamate to α–ketoglutarate (81). Mounting evidence suggests that the protein levels of KGA and GAC are controlled by c-MYC expression, promoting glutamine catabolism (82). Glutamine metabolites, namely, glutamic acid, α-ketoglutaric acid, and aspartic acid, can mediate the metabolism, epigenetic, nucleotide synthesis, and redox balance of tumor cells. Hence, a variety of compounds targeting glutamine metabolism have been developed for anticancer therapy (47).

The metabolism of branched-chain amino acid (leucine, isoleucine, and valine) affects diverse biological processes in protein synthesis and epigenetic regulation. The metabolic pathways of branched-chain amino acids are altered in many solid tumors, such as melanoma, NPC, and breast cancer (76, 83). Phosphoserine transaminase 1 (PSAT1), with a role in serine biosynthesis, is associated with NPC outcomes (84).

**Figure 3:**
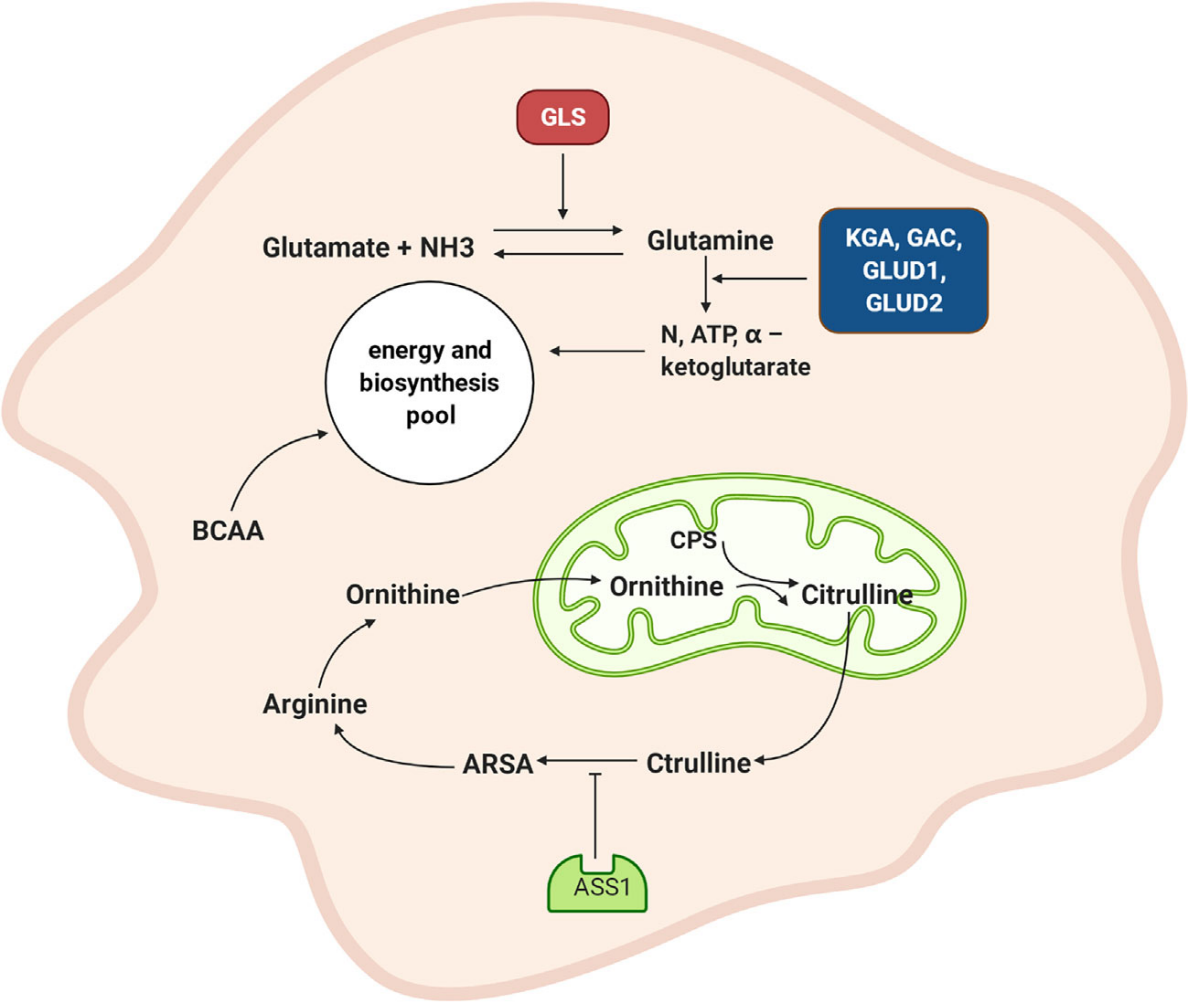
Amino acid metabolism of NPC cell. GLS promotes the combination of glutamate and amino groups to produce glutamine, which not only supplies energy but also scavenges ammonia. The expression of KGA and GAC subtypes are increased, and GLUD1 and GLUD2 are also up-regulated and promote the decomposition of glutamine and glutamic acid and produced more ammonia and α – ketoglutarate. This provides raw materials for energy generation and biosynthesis. ASS1 is down-regulated, which inhibited the ornithine cycle. GLS: glutamine synthetase, KGA: K-glutaminase GAC: glutaminase C, GLUD1: glutamate dehydrogenase 1, GLUD2: glutamate dehydrogenase 2, CPS: carbamoyl phosphate, ARSA: arginine succinate, ASS1: arginine succinate synthase, BCAA: branched-chain amino acid.

In summary (**Figure 3**), the synthesis and catabolism of glutamine play very prominent roles in NPC cells. Glutamine synthetase promotes a combination of glutamate and amino acids to form glutamine, which provides energy and scavenges ammonia. The upregulated expression of the KGA and GAC isoforms GLUD1 and GLUD2 promotes glutamine and glutamate decomposition and generates increasing levels of ammonia and α-ketoglutarate, which provides additional raw materials for energy generation and biosynthesis. When the ammonia pathway is blocked, more nitrogen becomes available. Downregulated ASS1 inhibits the ornithine cycle and boosts tumor cell proliferation. The metabolic pathway of branched-chain amino acids in NPC is dysregulated, although the detailed mechanism needs to be further explored.

**Figure 4:**
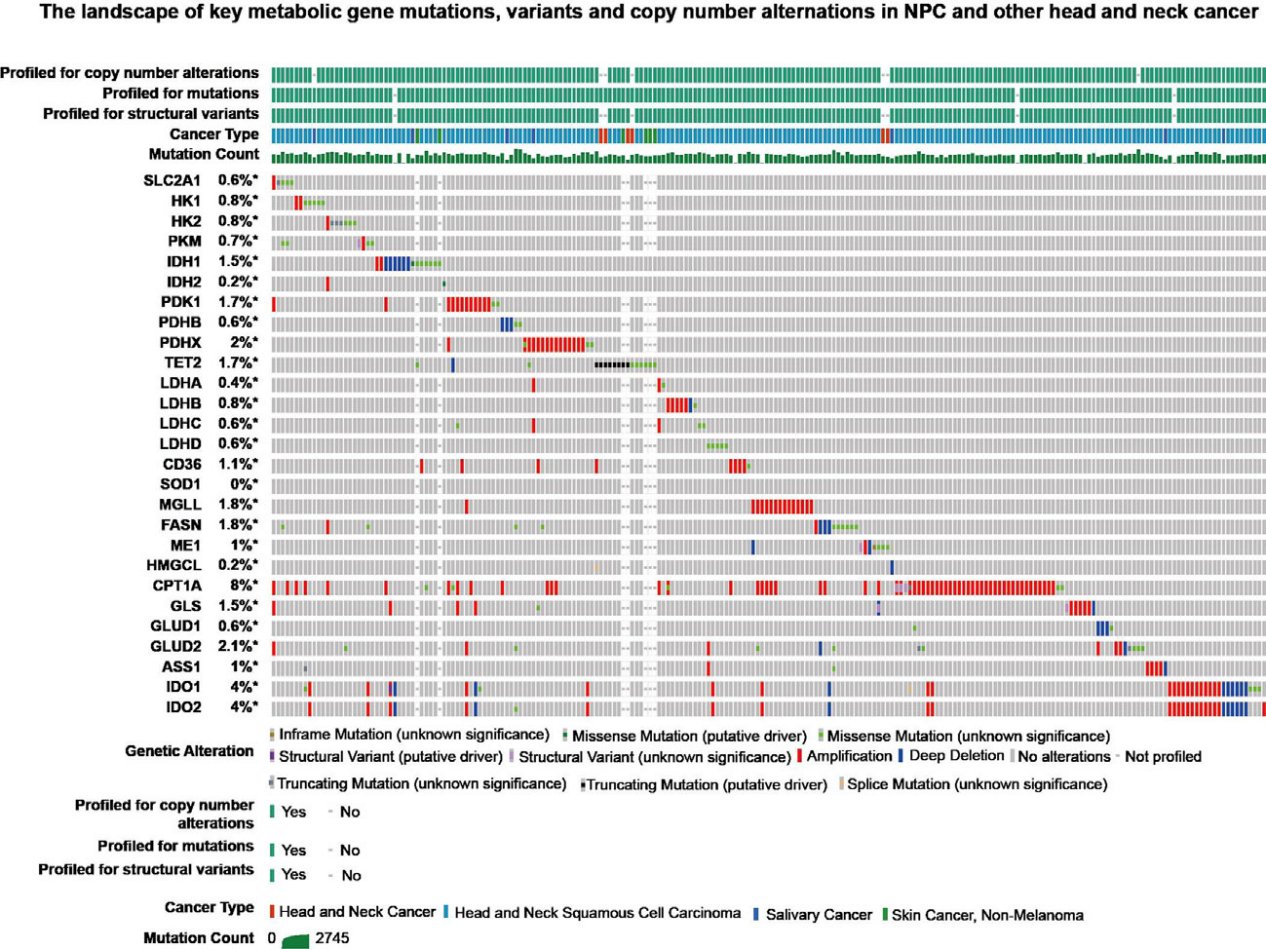
The landscape of key metabolic gene mutations, variants and copy number alternations in NPC and other head and neck cancer from cBioPortal (https://www.cbioportal.org/).

In three important metabolic pathways, key genes generally induce changes in metabolic processes. In order to further explore these genes, retrieval for transcriptional sequencing and somatic mutation was performed. A summary of the expression deregulation and somatic mutation of certain genes is presented in **Tables 2** and **3**, respectively. Overview of somatic mutation for key metabolic genes in NPC and other head and neck cancer is exhibited in **Figure 4**.

**Table 2:** The Expression of the Key Metabolic Genes in NPC Microarrays.

Genes	Expression
Glucose metabolism	
GLUT1	Upregulation^1^
HK2	Upregulation^1^
PKM2	Upregulation^1^
PDK1	Upregulation^1,2,3^
PDHB	Downregulation^1^
TET2	Upregulation^2^
LDHA	Upregulation^1^
LDHB	Upregulation^2^
LDHC	Upregulation^1^
IDH1	Upregulation^1,2^
IDH2	Upregulation^2^
G6PD	Upregulation^2^
Fatty acid metabolism	
SOD1	Upregulation^1,2,3^
PROX1	Upregulation^1,2^
FASN	Upregulation^1,2^
ME1	Upregulation^2^
CPT1A	Upregulation^2^
Amino acid metabolism	
ASS1	Downregulation^3^
Tryptophan metabolism	
IDO1	Upregulation^1^

Microarrays: ^1^E-GEOD-53819, ^2^E-GEOD-39826, ^3^E-GEOD-34573. All of microarrays were retrieved from ArrayExpression (https://www.ebi.ac.uk/arrayexpress/).

**Table 3:** Characteristics of Somatic Mutation of the Key Metabolic Genes in NPC.

Genes	Mutation ID	Genomic DNA Change	Type	Consequence type
GLUT1	MU121352410	Chr1:g.43395598C>A	Single base substitution	Stop Gained, Downstream, Intron
HK1	MU121336128	Chr10:g.71142451C>T	Single base substitution	Missense, Exon
HK2	MU121363760	Chr2:g.75115145A>G	Single base substitution	Missense
	MU121361238	Chr2:g.75115097A>-	Deletion of <=200bp	Frameshift
	MU5695667	Chr2:g.75115135G>A	Single base substitution	Synonymous
PDHX	MU121348454	Chr11:g.34938255T>A	Single base substitution	Missense, Upstream, Intron
PDHB	MU121351085	Chr3:g.58416566C>T	Single base substitution	Missense, Splice, Region,Exon,Downstream
IDH2	MU121345506	Chr15:g.90628519C>A	Single base substitution	Upstream, Synonymous, 3-UTR, Downstream
	MU121353948	Chr15:g.90630379T>C	Single base substitution	Missense, Upstream, 3-UTR, Downstream
FASN	MU121339044	Chr17:g.80041955A>G	Single base substitution	Missense, Upstream, Downstream
		chr17 80042195G>A	Single base substitution	Missense
		chr17 80039902C>A	Single base substitution	Missense
HMGCL	MU121332667	Chr1:g.24134708G>A	Single base substitution	Stop Gained, Splice Region, 3 UTR, Exon, Downstream
	MU121335427	Chr1:g.24134749C>-	Deletion of <=200bp	Frameshift, 3-UTR, Exon, Downstream, Intron
GLUD1		chr10 88818947C>G	Single base substitution	Missense
ASS1	MU121351353	Chr9:g.133327407G>-	Deletion of <=200bp	5-UTR,Upstream,Downstream, Intron

Somatic mutants of key genes were retrieved from ICGC Data Portal (https://dcc.icgc.org/) and the National Center for Biotechnology Information Sequence Read Archive (www.ncbi.nlm.nih.gov/sra; accession nos. SRA288429 and SRA291304).

## Reprogramming of Other Metabolic Pathways

### Nitric Oxide

In addition to the abovementioned metabolic patterns, NPC exhibits other metabolic reprogramming mechanisms. The central role of NOS is catalysis of the arginine precursor into nitric oxide (NO). Increased NO release has been detected in the tumor cells of recurring NPC (85, 86). The role of NO in inducing p53 mutation and angiogenesis has been established. Interestingly, tumor cell infiltration by NO can lead to the upregulation of DNA-dependent protein kinase catalytic subunit (DNA-PKcs); however, DNA-PKcs is typically directed to the repair of double-strand DNA breaks. Increased expression of DNA-PKcs protects cancer cells from the toxic effects of NO and the consequences of DNA damage (such as DNA-damaging chemotherapeutic drugs and X-ray irradiation) (87). In contrast to NO derived from tumor cells, NO derived from macrophages shows latent cytotoxicity to adjoining tumor cells (88).

Currently, radiotherapy is a fundamental treatment for NPC. Radiation-resistant tumor cells lead to residual and recurring cancer (89, 90). In radiation-resistant NPC cells, the production of NO is increased by upregulating the expression of CLIC4 (chloride ion intracellular channel 4), promoting the nuclear translocation of iNOS, reducing ROS levels, and inhibiting the cell death induced by ROS oxidative stress (85, 91). The internal tumor environment itself is anoxic (92). According to these characteristics, it can be hypothesized that radiation-resistant NPC cells create a stable microenvironment and acquire tolerance to stress. In general, NO production is increased in NPC, especially in radiation-resistant cancer cells. In the tumor microenvironment infiltrated by NO, DNA-PKcs is overexpressed in tumor cells, strengthening the resistance of tumor cells to NO toxicity and reducing tumor sensitivity to radiotherapy and chemotherapy on DNA damage.

### Fe Metabolism

Lactoferrin is an iron-binding protein, and its N-terminus is a serine protease with an immunological effect against bacteria and viruses (93). However, downregulated lactoferrin has been found to confer immune defence against EBV infection and to increase PDK1-induced glycolysis (94, 95). Furthermore, lactoferrin can inhibit NPC development and metastasis through the MAPK and PI3K/AKT pathways (94, 96).

Ferroptosis is a cell death mode that differs from apoptosis, necrosis, and autophagy and is caused by iron-dependent oxidative damage and associated with cytoplasmic and lipid peroxidation, smaller mitochondria, and higher mitochondrial membrane density (97). Ferroptosis is inhibited in the absence of glutamine or glutamine catabolism blockade. The decomposition product of glutamine, α-KG, is necessary for ferroptosis induction (98). The speed of glutathione synthesis is limited by cysteine level. Early studies found that exogenous cysteine is essential for the growth of HeLa and other cells (99). Cells cultured in cysteine-deficient medium die of glutathione depletion, which can be recovered by supplementing the medium with iron chelators (100, 101). Hence, ferroptosis depends on amino acid metabolism.

Iron is a trace element necessary for the accumulation of lipid peroxide and induction of ferroptosis. Transferrin and transferrin receptors, which enable iron internalization from the extracellular environment, are necessary for ferroptosis (98, 102). A low level of BDH2 (3-hydroxybutyrate dehydrogenase 2) amplifies NPC cell malignant behaviours by promoting intracellular iron overload, indicating that reduced intracellular iron might be favourable for NPC treatment (103). Fe is a cofactor in a variety of intracellular synthesis or decomposition reactions, and it contributes to ferroptosis induction and decreased NPC cell proliferation (104).

### Epigenetic Changes in Nucleotide Metabolism, DNA, and Histones

Nucleotide metabolism refers to the synthesis and catabolism of purine nucleotides and pyrimidine nucleotides, whose *de novo* synthesis requires glutamine, aspartic acid, one-carbon units of tetrahydrofolic acid, and CO_2_ (105). The expression of thymine synthase (TYMS) and dihydrofolate reductase (DHFR) in NPC is remarkably increased (106), which can lead to increased tetrahydrofolate expression for rapid nucleotide synthesis and to promoted pyrimidine nucleotides for facilitated tumor DNA replication and repair.

Epigenetic changes, especially DNA methylation abnormalities caused by EBV, play important roles in EBV-associated cancers such as NPC (107). DNA methylation relies on S-adenosylmethionine (SAM) to provide methyl groups, and the formation of SAM depends on extracellular folic acid, which can be increased in NPC cells by α-folate receptor activity (108). Isocitrate dehydrogenase (IDH1) mutation is associated with increased histone and DNA methylation because it decreases α-KG and α-KG-dependent prolyl hydroxylase (PHD) activity and increases HIF-1α activity (109–111). The expression of hypermethylated genes such as UBE2L6 is silenced in NPC, and this silencing has been found to stimulate ATGL degradation in adipocytes, causing abnormal catabolism (112). Similarly, ASS1, a rate-limiting enzyme in the ornithine cycle, is downregulated in NPC due to DNA methylation and contributes to lymphatic metastasis and invasion of malignant cells (77).

### The Relationship Between Three Major Metabolic Pathways

Glucose, lipids, proteins, and nucleic acids do not function in isolation; they are interrelated and transformed by common metabolic intermediates, the TCA cycle, and biological oxidation in NPC cells. In each metabolic process, the activity of enzymes is regulated by mutant genes or anomalous signalling pathways. For example, activation of the mutant p53 gene and the MAPK, Wnt/β-catenin, and PIK3/AKT signalling pathways primarily involves three major metabolic processes (113). Metabolic intermediates can prevent tumor cell apoptosis by affecting the levels of free radicals or ROS. Notably, in addition to regulating enzymes, these intermediates can be induced to modulate nutrient intake and transportation. The increased intake of glucose, fat, and protein meets the fast-growing energy needs of tumors. Amino acid synthesis, such as glutamine, provides substrates for purine and pyrimidine nucleotides. Fatty glycerol can be converted into dihydroxyacetone phosphate. Amino acids (alanine, tryptophan, serine, etc.) can be converted into pyruvic acid, which enhances glycolysis to a certain extent, produces lactate and provides energy quickly. NADPH provides acetyl-CoA with the reducing equivalents necessary for fatty acid synthesis (19).

## NPC Immune Evasion

Tumor cells resist or escape immune clearance by the host immune system through various mechanisms (114). The NPC tumor microenvironment (TME) is complex due to enriched lymphoid tissues and direct connection with the natural respiratory channels. Metabolic reprogramming involves metabolites and abnormally expressed metabolism-related proteins to cause immunosuppression. Here, we elaborate on the relationship between metabolic reprogramming and tumor-related immune escape.

**Figure 5:**
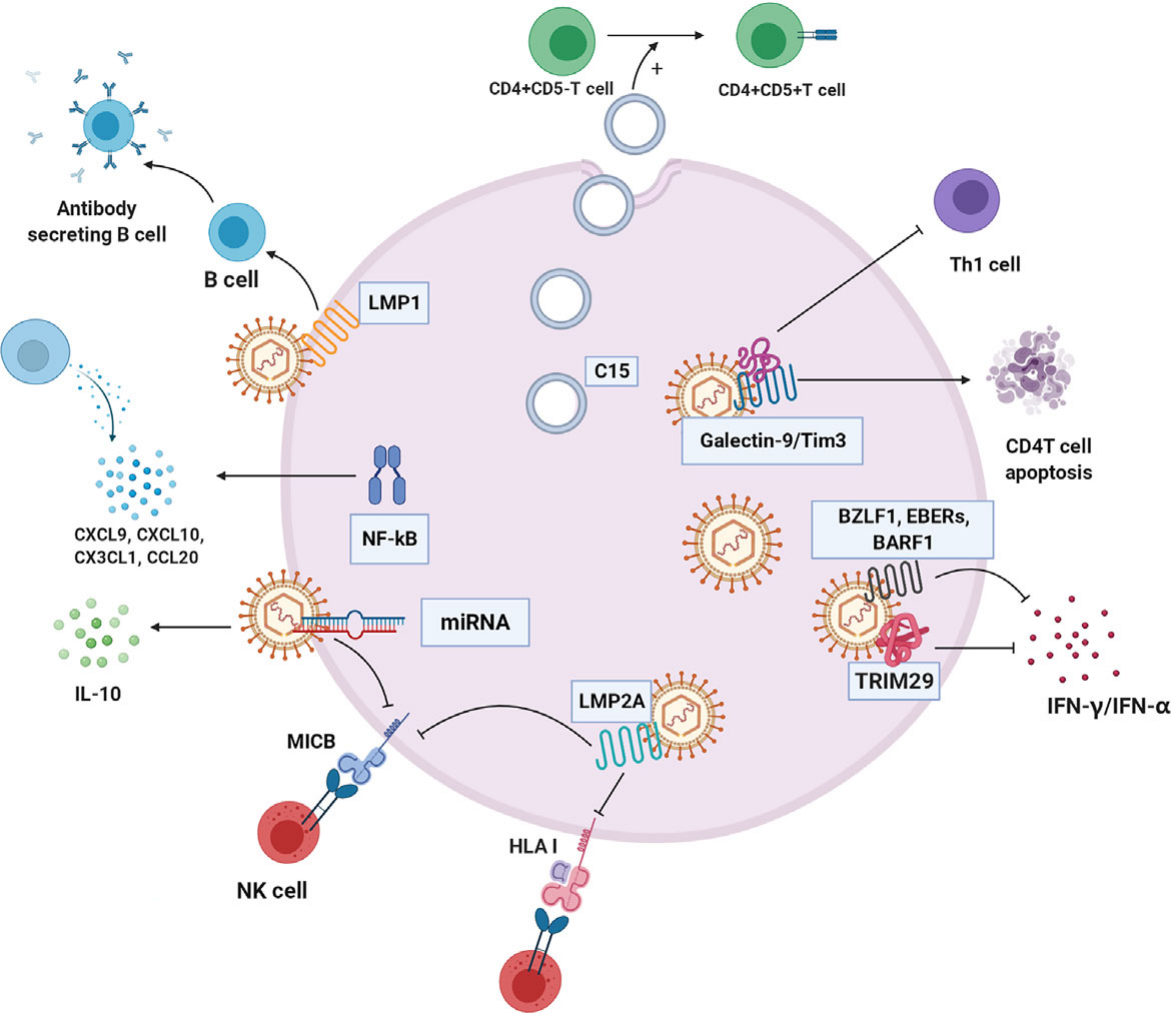
The immune evasion mechanism of NPC cell. C15 exosomes induced transformation and aggregation of CD4+CD25+Treg cells. NF-κB regulates a variety of chemokines CXCL9, CXCL10, CX3CL1, and CCL20 to reshape the tumor immune microenvironment. TRIM29 blocks interferon production. Galectin-9/Tim3 can induce apoptosis of CD4 T cells and inhibit Th1. LMP2A changes HLA-I, block recognition and presentation of cancer cell antigen peptide, and inhibit killing effect of NK cells. EBV-related products, such as BZLF1, EBERS AND BARF1, inhibit IFN - γ and IFN - α to escape immune clearance, respectively. LMP1 inhibits the differentiation of B cells into antibody-secreting cells. Some microRNAs of EBV down-regulate immune ligand MICB, which hinders recognition of NK cells.C15:C15 exosomes, CXCL9, CXCL10, CX3CL1, CCL20: chemokines of different subfamilies, TRIM29: EBV-induced tripartite motif-containing 29 protein, LMP2A: EBV latent membrane protein 2A, HLA-I: human leukocyte antigen I, BZLF1, EBERs, BARF1:EBV-related production, LMP1: EBV latent membrane protein 1, MICB: MHC class I chain-related MIC.

The coexistence of tumor-infiltrating lymphocytes and EBV-infected NPC cells creates a unique TME that supports immune escape in the early stage of EBV infection (115). EBV-infected NPC cells can secrete cytokines and exosomes containing viral products to modulate the function of stromal cells in the NPC tumor microenvironment and thus facilitate disease progression and prevent host immune attacks (1). Next, we describe the immune evasion mechanism of NPC cells (**Figure 5**).

### Exosomes

NPC cells imbue exosomes with LMP1, viral miRNAs, C15, and other molecules and secrete them into the immune environment (116). NPC-derived exosomes carrying C15 can transform CD4^+^CD25^-^ T lymphocytes into Treg cells (CD4^+^CD25^+^) and stimulate Treg cell aggregation and immunosuppressive effects, allowing tumor cells to escape immune clearance (117). Interestingly, studies have indicated that this immunosuppressive mechanism probably can be unleashed by treatment with an anti-CCL20 blocking monoclonal antibody (117, 118). LMP1 in exosomes effectively stimulates MAPK and Akt activation, promotes cell proliferation and inhibits host immunity (119). In addition, LMP1 can induce B cell activation but severely inhibits B cell differentiation into antibody-secreting cells (ASCs) (120).

### Cytokines and Chemokines

EBV disrupts the immunological response by stimulating regulatory T cells that secrete IL-10, an inhibitory cytokine that reduces the infiltration of cytotoxic T cells (121, 122).

NPC is abnormally regulated through four cancer-related pathways (the EGFR-PI3K-Akt-mTOR, NOTCH, and NF-κB pathways) (123). EBNA1, a gene encoded by EBV, inhibits NF-κB activity in the epidermis, a situation that can be exploited for immune escape and can cause cells to be continuously infected (124, 125). In contrast, LMP1 is an effective activator of the NF-κB signalling pathway (126). In EBV-positive NPC cells, activated NF-κB regulates the production of many chemokines, such as CXCL9, CXCL10, CX3CL1, and CCL20, leading to T lymphocyte infiltration into the cancer environment of NPC (1). Regardless, certain viral oncoproteins lead to the stimulation of the NF-κB pathway; the constitutive activation of NF-κB may be part of an important mechanism by which tumor cells escape immune surveillance and resist immunotherapy (127).

EBV induces members of the TRIM29 protein family to inhibit innate immunity in human airway epithelial cells. TRIM29 knockout accelerates type-I interferon (IFN1) and almost eliminates EBV and lethal herpes simplex virus-1 in human NPC cells (128). Therefore, EBV and possibly other double-stranded DNA viruses drive TRIM29 to suppress local innate immunity, resulting in persistent DNA viral infection. In addition, exosomes carrying galectin-9 released by EBV-infected NPC cells inhibit Th1 activity and induce the apoptosis of EBV-specific CD4 T cells (114). Blocking galectin-9/Tim-3 activity *in vitro* can release Th1 inhibition and lead to a maintained antitumor T cell response, thus improving the clinical efficacy of NPC immunotherapy (129).

### Mutation of HLA Epitopes Associated With EBV

EBV strains carrying LMP1 and HLA-A2-restricting epitopes are prevalent in NPC in populations in South China and Taiwan (130). This EBV strain resists immune recognition, evades CTL recognition, weakens the IFN-γ response, and may lead to NPC prevalence to some extent (131). LMP2A downregulates the expression of HLA-ABC and MIC-A/B through promoter hypermethylation, hinders recognition and presentation of endogenous antigenic peptides, and inhibits T and NK cell action (132).

### Products Related to EBV

EBV affects interferon expression to support EBV-positive NPC growth *in vivo* (133). The early EBV protein BZLF1 decreases IFN-γ receptor expression, which further affects IFN-γ-induced MHC-II expression, implying that EBV can evade the antiviral immune response in early infection (134, 135). Another EBV secretory protein, BARF1, directly inhibits α-interferon secretion from monocytes, reducing the innate response to the virus (136). Similarly, BDLF3, encoded by EBV, is an early factor that can target the cell surface and intracellular MHC-I molecules for ubiquitin-mediated proteasome degradation and induce immune cell apoptosis (137).

LMP1 not only affects NPC cell metabolism but also participates in host antitumor immunity. LMP1-mediated glycolysis regulates IL-1β, IL-6, and GM-CSF expression through the NLRP3 inflammatory body, COX-2, and NF-kB signalling pathways, which enhances the expansion of tumor-related bone marrow-derived suppressor cells (MDSCs) (138).

The immune evasion of cancer is also related to virus-related microRNAs (139). In many viruses, such as HCMV and EBV, miRNAs target the immune ligand MICB and downregulate MICB, resulting in inhibited recognition of natural killer cells (140). In addition, EBV miR-BART15 decreases IL-1β expression (141).

### Effects of Metabolites Derived From Tumor Cells on Immune Cells

Lactate is the metabolite in cancer cells with the most profound effect. A high concentration of lactate interferes with T cell metabolism (142), damages dendritic cell and tumor-associated macrophage function, and inhibits monocyte migration and cytokine release, which promote cancer cell development (143–145). Lactate mediates M2-like polarization of tumor-associated macrophages (TAMs) through hypoxia-inducible factor-1α (HIF-1α), activates the VEGF pathway and augments cancer cell perfusion in blood (146–148). Acidification of the tumor environment caused by lactate inhibits monocyte-secreted TNF, protecting malignant cells from immune clearance (149).

In addition to immunosuppression by environmental acidification, other tumor metabolites, such as adenosine, kynurenine, and tryptophan, can play roles in tumor immunity. Extracellular adenosine weakens the adhesion and cytotoxicity of T cells *via* A2a and A3 adenosine receptors (150). Indoleamine-2,3-dioxygenase (IDO) is highly expressed in tumor cells, catalysing tryptophan, which negatively regulates the immune response, inhibits T cell proliferation, and induces T cell apoptosis, thus causing immune tolerance (151, 152). As shown in **Table 2**, IDO1 is overexpressed in NPC. Kynurenine catabolized from tryptophan within tumor cells can promote Treg cell function and numbers by activating kynurenine aryl-hydrocarbon receptor (AHR) in CD4^+^ T cells (47). Furthermore, IDO directly activates Treg cells. IDO inhibitors are being actively studied as single agent drugs or for use in combination with other treatments against cancer (153–155). Lipids obtained in part from adjacent cancer cells with enhanced fatty acid synthesis accumulate in tumor-infiltrating myeloid cells, including myeloid-derived suppressor cells (MDSCs) and TAMs, and can promote metabolic reprogramming and promote the acquisition of immunosuppressive phenotypes (47). MAGL induces TAMs to M2-like polarization, helping tumor cells evade immune clearance (156).

## Nutritional Competition Between Cancer Cells and Immune Cells

**Figure 6:**
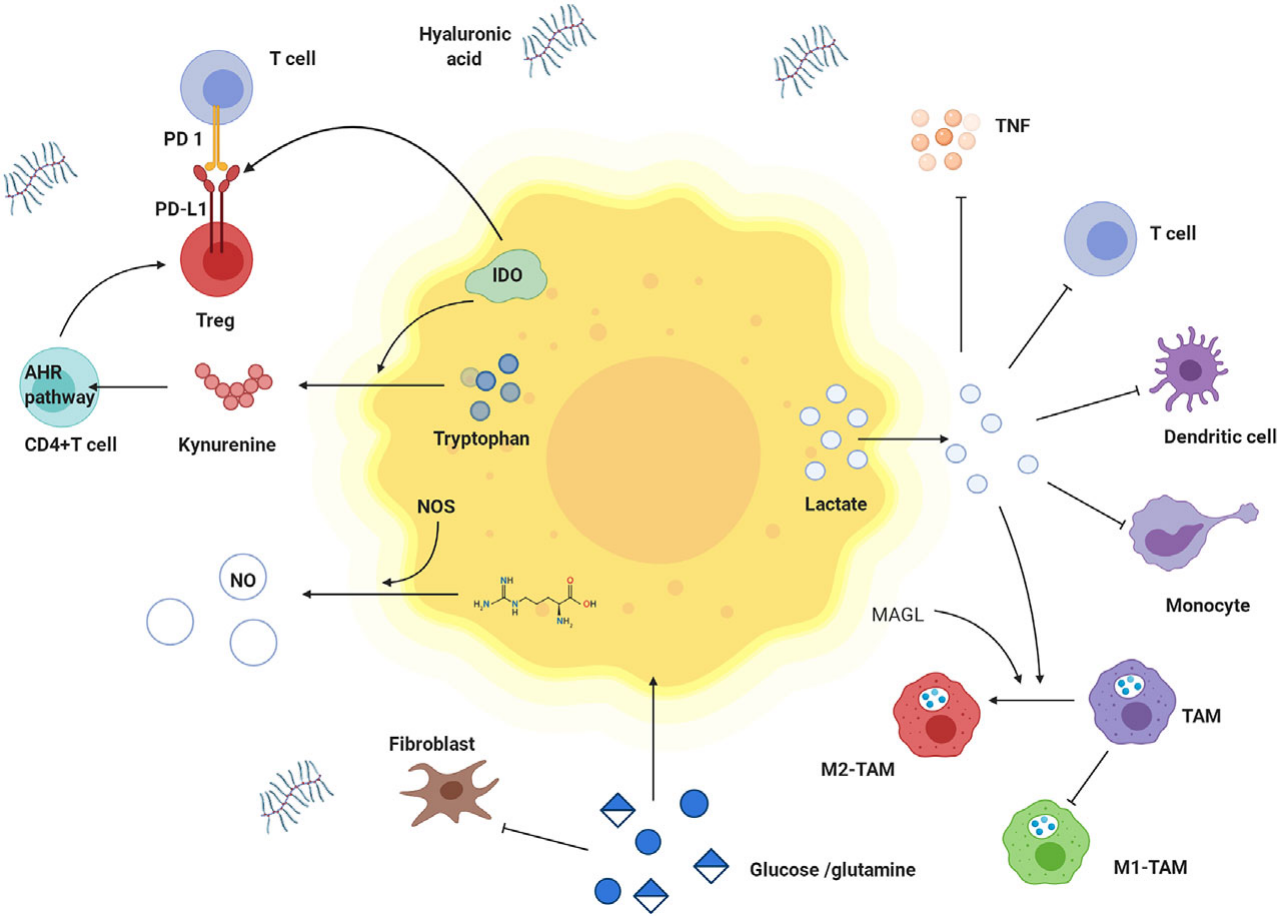
Effects of metabolites derived from tumor cells on immune cells and nutritional competition between cancer cells and immune cells. Tumor cells have competitive advantages over glutamine and glucose. Tumor cells produce a lot of lactate, which is transported to extracellular environment. High concentration of lactate hinders transfer of lactate from T cells and interferes with metabolism and function of T cells. Lactate damages dendritic cells and inhibits monocyte migration and cytokine release. Lactate inhibits the effect of TNF. In tumor microenvironment, tryptophan is decreased, while kynurenine and tryptophan is increased. IDO is highly expressed in tumor cells and catalyzes tryptophan degradation. Tryptophan is metabolized to produce kynurenine, which promotes the differentiation of CD4 + T cells into Treg cells by activating kynurenine AHR signal axis. IDO also activates Treg cells to up-regulate the expression of PD-L1 and inhibited the proliferation of T cells through PD-1/PD-L1 pathway. M1-TAM decreases but M2- TAM increases. Lactate and MAGL induced M2 like polarization of TAMs. In addition, hyaluronic acid is increased in matrix, and Treg cells are highly expressed. TNF: tumor necrosis factor, IDO: indoleamine 2,3-dioxygenase, AHR pathway: kynurenine aryl hydrocarbon receptor signal axis, TAM: tumor-associated macrophage, M1-TAM: M1 type tumor-associated macrophage, M2-TAM: M2 type tumor-associated macrophage.

Energy tumor cells and immune cells compete within the tumor microenvironment. Metabolic reprogramming helps tumor cells increase their competitive advantage by enabling greater absorption of nutrients and acquisition of energy than their surrounding immune cells, which greatly weakens the tumor cell recognition and clearance by host immunity (157). Glutamine deprivation in cancer cells inhibits T cell proliferation and cytokine production (47). The increased utilization of glucose in tumor cells leads to limited extracellular levels for use by tumor-infiltrating immune cells. Similarly, upregulation of GLUT1 expression in NPC cells accelerates glucose consumption, which inhibits T cell effector functions. Thus, the T cell response against NPC might be restored by blocking cancer cell glucose and arginine consumption and increasing their levels in infiltrating T cells (49). These two aforementioned mechanisms are depicted in **Figure 6**.

## Progress of Immunotherapy and Metabolism-Targeted Therapy in NPC

Recently, increasing evidence has shown that anti-PD-1 and anti-PD-L1 monoclonal antibodies are effective in the treatment of head and neck squamous cell carcinoma, especially for recurrent or metastatic head and neck squamous cell carcinoma (158–162). Treating NPC patients with PD-L1 blockade is an effective strategy (163, 164); however, the anti-PD1 monoclonal antibody induces an immune response in NPC patients but shows no substantial effect in reducing tumor burden (165).

Clinical studies have suggested that EBV-specific cytotoxic T cell (EBV-CTL) infusion is safe and effective because it targets the EBV antigens EBNA1 and LMP1 and LMP2 (166). Interestingly, Fas gene silencing in EBV-CTL cells might facilitate cytotoxic T cells clearance of NPC cells (167). Given that almost all EBV-positive nasopharyngeal carcinomas encode LMP1, the Ad-Δ LMP1-LMP2 gene was used to transduce dendritic cells in patients with advanced nasopharyngeal carcinoma and achieved satisfactory results in a clinical trial (168). However, immunotherapy alone may have only limited effects on constrained cancers. Small-molecule drugs or antibody interventions targeting metabolic processes can be used as pharmacological adjuncts with checkpoint blockade, as suggested by their roles as reported in preclinical models and ongoing or closed clinical trials (summarized in **Table 4**). Metabolic inhibitors such as 3-bromopyruvate and oxamate have been reported to suppress HK and LHDA activity in tumors and promote macrophage and T cell activation in a preclinical NPC model (22, 40). Other metabolic inhibitors, including 2-DG, dichloroacetate (DCA), and epacadostat, have entered or passed different stages of clinical trials, showing promising therapeutic effects on metabolic targets by reinforcing T cells and suppressing tumor metabolism. Given the impacts of metabolism on immunity as mentioned above and the history of therapy development, the combination of metabolic drugs with immunotherapy reagents may help overcome immunotherapy resistance and may soon be a potential clinical option for cancer patients.

**Table 4:** Metabolic Inhibitors with Reported Immunomodulatory Roles in Cancer.

Metabolism pathway	Target-based actions	Drug name	Condition or disease	Clinical trials	Potential immunomodulatory roles for immune cells
**Glucose metabolism**	HK inhibitor	3- Bromopyruvate	NPC (22) MPNST (169)	NA	Modulate Th17/Treg cell differentiation and suppressing dendritic cell (170); alter cell survival and repertoire of TAM (171)
		2-DG	Advanced cancer including prostate cancer and HNSCC	Phase I/II (NCT00633087) Phase I (NCT00096707)	Enhance CD8+ T cell memory and anti-tumor function (172)
	PDHK1 inhibitor	Dichloroacetate (DCA)	Metastatic Breast cancer, lung cancer and HNSCC	Phase II NCT01029925) Phase II (NCT01386632) Phase I (NCT01163487)	Induce regulatory T-cell differentiation and suppresses Th17-cell differentiation (173); induce favors differentiation towards the regulatory T-cell subset instead of effector T-cell subsets (174)
	PKM2 inhibitor	Shikonin	Breast cancer Bladder Urothelial cancer	NCT01287468 NCT01968928	Induce immunogenic cell death and enhance dendritic cell (175)
	LDHA Inhibitor	FX11	Pancreatic cancer (176)	NA	Inhibit proliferation of CD8+ T cells, and CD8+ T cell effector functions (177)
		Oxamate	NPC (40) NSCLC (178)	NA	Induce T cell activation (179)
**FAS**		TVB-2640	Solid cancer colon cancer breast cancer	Phase I (NCT02223247) Phase I (NCT02980029) Phase II (NCT03179904)	Inhibit CD8+ T cell and TH17 cell differentiation and function; increases Treg cell differentiation (180)
		C75	Prostate cancer (181)	NA	Protect effector CD4 T cells derived from naïve, effector memory, and central memory T cell subsets (182)
	CPT1A	Etomoxir	Ovarian Cancer (183)	NA	Affect T cell proliferation (184)
	GLS	CB-839	NSCLC, RCC, melanoma; NSCLC; solid cancer	Phase I/II (NCT02771626) Phase II (NCT04265534) Phase I/II (NCT03965848)	Activate tumor antigen-specific T cells and improve tumor-killing activity (185)
**Glutaminolysis**		BPTES	Cancer (186)	NA	Decrease Th17 differentiation (187)
	Glutamine requiring enzymes	DON	Cancer (188)	NA	Inhibit the differentiation of dendritic cells and macrophages (189)
**Glutamine metabolism**	Arginase inhibitor	CB-1158	Metastatic cancer	Phase I (NCT02903914) Phase I(NCT03837509)	Increase tumor-infiltrating CD8+ T cells and NK cells, inflammatory cytokines, and expression of interferon (190)
**R-2- HG synthesis**	IDO inhibitor	Epacadostat	HNSCC Metastatic NSCLC	Phase I (NCT03358472) Phase III (NCT03342352) Phase II (NCT03322540)	Reduce numbers of IDO1-expressing myeloid-derived suppressor cells (191)
**Tryptophan metabolism**		Indoximod	Metastatic prostate cancer	Phase II (NCT01560923)	Recruit cytotoxic T lymphocytes, reduce Foxp3+ T cells (192)
	Adenosine receptor A2A	PBF-509	NSCLC	Phase I/II (NCT02403193)	Increase responsiveness of CD4+ T cell (193)
		AZD4635	Advanced solid cancer prostate cancer	Phase I (NCT03980821) Phase II (NCT04495179)	Decrease expression of PD-1 and LAG-3 on both CD8+ effector T cells and Tregs (194)

GLUT1, glucose transporter; HK, hexokinase; MPNST: malignant peripheral nerve sheath tumor; TAM, tumor associated macrophages; 2-DG, 2- deoxyglucose; PDHK1, pyruvate dehydrogenase kinase; HNSCC, squamous cell carcinoma of head and neck;HCC, hepatocellular carcinoma; PFKFB3, 6- phosphofructo-2- kinase/fructose-2,6- biphosphatase; GVHD, graft-versus-host disease; GAPDH, glyceraldehyde 3- phosphate dehydrogenase; PKM2, M2 pyruvate kinase; LDHA, lactate dehydrogenase A; NSCLC, non-small cell lung cancer; TMJOA, temporomandibular joint osteoarthritis; G6PD, glucose-6- phosphate dehydrogenase; FAS, fatty acid synthesis; ACC1, acetyl- CoA carboxylase; FAO, fatty acid β-oxidation; CPT1A, carnitine palmitoyl transferase 1; AML, Acute myeloid leukemia; GLS, glutaminase; BPTES: bis-2-(5-phenylacetamido-1, 3, 4-thiadiazol-2-yl) ethyl sulfide; DON: 6-diazo-5-oxo-L-norleucine; IDH1, isocitrate dehydrogenase; IDO, indoleamine 2, 3- dioxygenase; IDO, indoleamine 2,3- dioxygenase; L-1-MT, 1-Methyl-L-tryptophan; NA, not applicable.

## Limitation and Perspective

Despite great growing reports on metabolic rewiring and immune escape in NPC-associated cancer research, most of them are limited to tell one side (either metabolic rewiring or immune escape) over both sides’ story. The mutual interactions between metabolic reprogramming and immune escape in NPC are crucial to understanding the comprehensive mechanism of treatment-resistant, which should be further investigated in the future. Although metabolic interventions that enhance anti-tumor immunity have been developed and trialed in cancers, few enroll NPC patients. Given the influence of EBV in NPCs, another direction could be studying the mutual connection of metabolic reprogramming with host immunity in an EBV-associated virologic environment.

## Author Contributions

Conceptualization: HH and GZ. Writing—original draft preparation: HH. Writing—review and editing: HH and GZ. Supervision: GZ, SL and QT. Funding acquisition: GZ and HH. All authors contributed to the article and approved the submitted version.

## Funding

This review was supported by National Natural Science Foundation of China (Nos. 82173341 and 81602389), Natural Science Foundation of Hunan Province (Nos. 2020JJ4827 and 2017JJ3456), Graduate Independent Exploration and Innovation Project of Central South University (Nos.2021zzts1069), and the Project of Hunan Health Commission (B2019165).

## Conflict of Interest

The authors declare that the research was conducted in the absence of any commercial or financial relationships that could be construed as a potential conflict of interest.

## Publisher’s Note

All claims expressed in this article are solely those of the authors and do not necessarily represent those of their affiliated organizations, or those of the publisher, the editors and the reviewers. Any product that may be evaluated in this article, or claim that may be made by its manufacturer, is not guaranteed or endorsed by the publisher.
